# Tumor-derived KLK8 predicts inferior survival and promotes an immune-suppressive tumor microenvironment in lung squamous cell carcinoma

**DOI:** 10.1186/s12890-023-02770-4

**Published:** 2024-01-25

**Authors:** He Tian, Ran Wei, Chu Xiao, Tao Fan, Yun Che, Tiejun Liu, Bo Zheng, Chunxiang Li, Jie He

**Affiliations:** 1 Department of Thoracic Surgery, National Cancer Center/National Clinical Research Center for Cancer/Cancer Hospital, Chinese Academy of Medical Sciences, Peking Union Medical College, Beijing, 100021 China; 2 Department of Colorectal Surgery, National Cancer Center/National Clinical Research Center for Cancer/Cancer Hospital, Chinese Academy of Medical Sciences, Peking Union Medical College, Beijing, 100021 China; 3 Department of Pathology, National Cancer Center/National Clinical Research Center for Cancer/Cancer Hospital, Chinese Academy of Medical Sciences, Peking Union Medical College, Beijing, 100021 China

**Keywords:** KLK8, Squamous cell lung carcinoma (LUSC), Prognosis, Tumor immune microenvironment (TIME), Biomarker

## Abstract

Lung squamous cell carcinoma (LUSC) is the second most common lung cancer worldwide, leading to millions of deaths annually. Although immunotherapy has expanded the therapeutic choices for LUSC and achieved considerable efficacy in a subset of patients, many patients could not benefit, and resistance was pervasive. Therefore, it is significant to investigate the mechanisms leading to patients’ poor response to immunotherapies and explore novel therapeutic targets. Using multiple public LUSC datasets, we found that Kallikrein-8 (KLK8) expression was higher in tumor samples and was correlated with inferior survival. Using a LUSC cohort (*n* = 190) from our center, we validated the bioinformatic findings about KLK8 and identified high KLK8 expression as an independent risk factor for LUSC. Function enrichment showed that several immune signaling pathways were upregulated in the KLK8 low-expression group and downregulated in the KLK8 high-expression group. For patients with low KLK8 expression, they were with a more active TME, which was both observed in the TCGA database and immune marker immunohistochemistry, and they had extensive positive relations with immune cells with tumor-eliminating functions. This study identified KLK8 as a risk factor in LUSC and illustrated the associations between KLK8 and cancer immunity, suggesting the potentiality of KLK8 as a novel immune target in LUSC.

**Supplementary Information:**

The online version contains supplementary material available at 10.1186/s12890-023-02770-4.

## Introduction

According to the newest data released in 2022, lung cancer causes the most cancer-related death annually worldwide [1], and lay a heavy burden on the public health system. Non-small cell lung cancer (NSCLC) takes up approximately 85% of lung cancer, and 25–30% of NSCLC patients can be classified into lung squamous cell carcinoma (LUSC) [2]. Unlike other NSCLCs, LUSC is featured by male gender, advanced age, smoking, and various comorbidities. In clinical practice, LUSC typically occurs in the central region of the lung parenchyma, and the 5-year overall survival (OS) rate of late-stage LUSC was dismal [3].

Currently, the main treatment strategy for LUSC patients is platinum-based chemotherapy, while there is a substantial of LUSC patients who could not benefit [4]. The targetable driver mutations of LUSC are few, limiting the application of targeted therapy in LUSC [5]. In the recent decade, immunotherapy has shifted the paradigm of cancer treatment and offers hope to LUSC patients who are refractory to traditional therapies [6]. However, durable clinical benefits are rarely achieved, and drug resistance and disease recurrence are prominent issues in LUSC immunotherapy [7]. Research focusing on optimizing immunotherapy in LUSC patients is warranted.

Kallikrein-related peptidases (KLKs) are a family of serine proteases containing 15 genes (KLK1 to KLK15) located on chromosome 19q13.3–13.4, they are closely associated with numerous pathophysiological processes, including cancer and infectious diseases [8, 9]. In cancer immunity, KLKs are critical mediators of the tumor microenvironment (TME), regulating multiple bioactive molecules, including the extracellular matrix architecture in tumor progression [10]. Proteins from the kallikrein family could mediate cancer progression through multiple signaling pathways, including the PAR (protease-activated receptors) signaling pathway, the IGF (insulin like growth factor) signaling pathway, the kallikrein signaling pathway, and the steroid hormone signaling pathway [8].

KLK8, one of the members of the KLKs family, is a synaptic, plasticity-modulating extracellular serine protease, involved with several malignant diseases, including ovarian cancer [11, 12], cervical cancer [13], breast cancer [14], colorectal cancer [15, 16], oropharyngeal cancer [17], gastric cancer [18], pancreatic cancer [16], and NSCLC [19–22]. In lung cancer, Sher et al. reported that KLK8 could suppress tumor invasiveness and thus relate to a favorable prognosis in NSCLC [21]. While Planque et al. found that KLK8-T3 and KLK8-T4, which were two alternative splicing variants of KLK8, were independent predictors of low survival in NSCLC [20].

Controversial views exist about the roles of KLK8 in lung malignancies and little is known about the associations between KLK8 and lung cancer immunity. Therefore, it is of significance to further clarify the role of KLK8 in NSCLC.

Here, we explored the prognostic ability and immune background of KLK8 in LUSC. We validated our bioinformatic findings in a large-scale LUSC cohort with long-term survival follow-up. Our work illustrated the prognosis capacity of KLK8 and shed light on the therapeutic possibility of KLK8 in LUSC.

## Materials and methods

### Pan-cancer expression analyses of KLK8

The expression of KLK8 of pan-cancer types was analyzed and visualized by the online tool TIMER (http://timer.cistrome.org/). The expression of KLK8 in the Cancer Genome Atlas (TCGA) LUAD and LUSC datasets was calculated and visualized by the online tool GEPIA (http://gepia.cancer-pku.cn/).

We used the ‘Cancer Exploration-Gene_DE’ module of TIMER. This module allows users to study the differential expression between tumor and adjacent normal tissues for any gene of interest across all TCGA tumors. Distributions of gene expression levels are displayed using box plots. The statistical significance computed by the Wilcoxon test is annotated by the number of stars (*: *p*-value < 0.05; **: p-value < 0.01; ***: p-value < 0.001). Users can identify genes that are up-regulated or down-regulated in the tumors compared to normal tissues for each cancer type, as displayed in gray columns when normal data are available. (http://timer.comp-genomics.org/timer/). The datasets that GEPIA used is based on the UCSC Xena project (http://xena.ucsc.edu), which is computed by a standard pipeline.

The public gene-expression data for transcriptome profiling and the corresponding clinical annotation were obtained from Gene Expression Omnibus (GEO) and the TCGA database on May 1, 2022. There were four eligible lung cancer cohorts of geneexpression data (GSE73403, GSE15011, GSE30219, TCGA-LUSC and TCGA- LUAD). We downloaded the raw microarray data form the Affymetrix Human Genome U133 Plus 2.0 Array of GEO database and the RNA sequencing data (fragments per kilobase of transcript million mapped reads (FPKM) value) of TCGA. We employed the “ComBat” algorithm in “SVA” package to adjust the batch effects from nonbiological technical biases among different LUSC and LUAD RNA-seq data. And all of the RNA-seq data were adjusted for background adjustment and quantile normalization with robust multiarray averaging method in “affy” and “simpleaffy” packages.

All lung cancer samples were coded according to the third Edition of the International Classification of Diseases for Oncology (ICD-O-3). Patients were included based on the following criteria: (1) Aged between 18 and 75 years; (2) lung cancer as the first cancer diagnosis, microscopically confirmed adenocarcinoma or squamous carcinoma. The exclusion criteria included patients with incomplete survival information and missing data on neoplasm histologic type.

### Survival analysis

The survival analyses were performed based on the patients from the TCGA LUSC cohort, GSE73403, GSE15011, GSE30219, and the LUSC cohort enrolled at National Cancer Center (Beijing, China), using the R package “survival” and “survminer” [23, 24]. The patients were stratified by the median expression value of KLK8 of each dataset.

### Tissue microarray construction and Immunohistochemical staining (IHC)

Under the IRB (Institutional Review Board) approval, formalin-fixed, paraffin-embedded (FFPE) tissue microarray (TMA) were constructed using LUSC samples collected from patients accepting surgery from April 2010 to September 2011 in the Department of Thoracic Surgery, Cancer Hospital, Chinese Academy of Medical Sciences and Peking Union Medical College. The TMA included 190 LUSC tumor samples. All specimens in the TMAs were diagnosed, selected, and confirmed by two certified pathological clinicians. For each sample, we took two 2-mm cores to constitute the TMAs, and then the 4-mm thick TMA sections were manufactured. All manual process was conducted by the technicians from the Department of Pathology of our hospital.

We performed the immunohistochemistry (IHC) of several markers on the TMA, including KLK8, PD-1, PD-L1, CD8, CD68, CD47, PVR, and TIGIT. We incubated the TMAs with the primary antibodies against KLK8 (Abcam, ab150395), PD-1 (CST, D4W2J), PD-L1 (Abcam, 28–8), CD8 (CST, D8A8Y), CD68 (Abcam, KP1), CD47 (Abcam, EPR21794), PVR (CST, D8A5G), and TIGIT (CST, E5Y1W), and then with the secondary antibodies and 3,3′-diaminobenzidine (DAB). Two independent pathologists blinded to our research evaluated the IHC staining results. KLK8 expression was scored using a combined method (21). Negative, weak, moderate, and strong intensities were scored as 0, 1, 2, and 3, respectively. The percentage of cells that were stained at each intensity score was estimated visually. The final score for each specimen was calculated as the sum of the percentage of stained cells multiplied by the intensity scores. For example, a sample with 20% negative staining, 40% moderate staining, and 40% strong staining would be assessed with a score of 2.3 (0.2 × 0 + 0.4 × 2 + 0.4 × 3 = 2.0). All samples were scored independently by two pathologists who were blinded to our study. For CD8 and CD68, we calculated the number of CD8-positive TILs and CD68-positive macrophages under six high-power fields and took the average for each specimen. For PD-L1 and CD47, we applied the membranous tumor proportion score (TPS), during which TPS ≥ 1% and TPS ≥ 5% were set as the positive standard for the two markers, respectively. PD-L1 and CD47 co-expression was defined as samples positive in both PD-L1 and CD47.

### Construction of prognostic prediction model

The correlation between clinicopathologic factors and patients’ survival was evaluated by univariate and multivariate Cox regression model. Based on the multivariate Cox regression model, the survival prediction model and nomogram were constructed and plotted by the R package “RMS”. The area under the curve (AUC) and the time-dependent receiver operating characteristic (ROC) curve were used to evaluate the prognostic accuracy of the nomogram model in different sets with the package “pROC”.

### Function enrichment analysis

The transcriptome dataset of the TCGA LUSC cohort was used for functional enrichment analysis. The gene set enrichment analysis (GSEA) for transcriptome data of two patient groups was performed by GSEA software (V.4.1.0), including C2 (KEGG) [25–27] and C5 (GO) gene sets, and NOM *p <* 0.05 and FDR *q <* 0.05 were considered as significant. The GO and KEGG pathway analyses for the differentially expressed genes (DEGs) between high-KLK8 and low-KLK8 patient groups were conducted and visualized by R packages “clusterProfiler”, “org.Hs.eg.db”, “enrichplot”, and “ggplot2”.

### Immune cell infiltration analysis

The infiltration score of various immune cells and stroma cells within the tumor microenvironment for low-KLK8 and high-KLK8 patient groups were calculated by XCELL, CIBERSORT, EPIC, and MCP-counter algorithm based on the transcriptome data of the TCGA LUSC dataset (Supplementary Data 1). The heatmap was plotted by the R package “pheatmap”. The correlation analyses between KLK8 expression (log_2_TPM) and the infiltration level of immune cells which were evaluated by different algorithms were conducted and visualized by the online tool of TIMER [28], R package “MCPcounter”, “e1071”, “preprocessCore”.

### Statistical analysis

The Spearman correlation coefficient was used to explore the associations between variables. Difference analyses for two groups were analyzed with two-tailed unpaired Student’s t-test, and three or more groups were analyzed with one-way ANOVA. The log-rank test and the Kaplan–Meier survival analysis were used to evaluate the difference in overall survival (OS) among different KLK8 expression level. The univariable and multivariate Cox regression analyses were used to identify the independent prognostic factors and to establish nomogram based on the forward and backward elimination methods. All clinicopathological factors and immune marker expression status were included. The nomogram evaluating the 1-year, 3-year, and 5-year survival data of each patient was displayed and the regression formula was “1.0677*T+ 0.9634*N+ 0.6733*CD47- CD8*0.3057+ PVR*0.8723+ KLK8*0.7273”. The patients were stratified by the median expression value of KLK8 of each dataset. Data were represented as mean ± SEM. *P* values < 0.05 was significant. Data were analyzed with the R (version 4.2.1) and GraphPad Prism 8.4.3.

## Results

### High KLK8 is associated with poor prognosis in LUSC

**Fig. 1:**
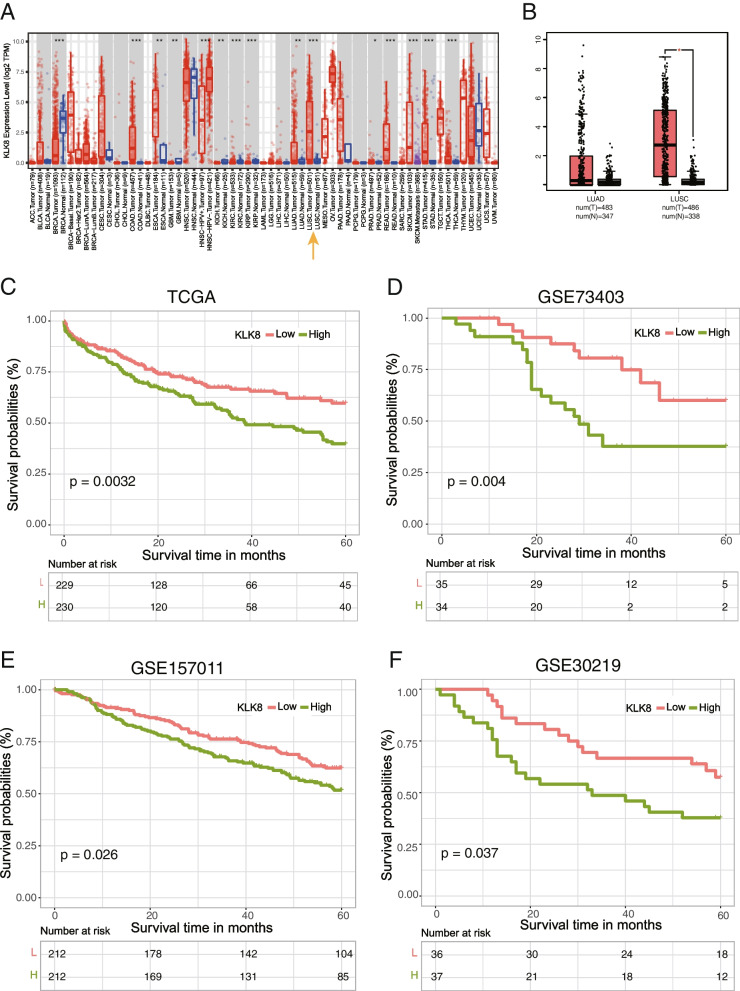
KLK8 expression in cancer and its correlation with survival. **a** KLK8 expression profiles in the tumor and paired normal tissues of pan-cancer types in TIMER. The height of the bar represents the median expression of certain tumor types or normal samples. Red, tumor; Blue, normal tissue. Yellow arrow, KLK8 expression in LUSC. ACC, Adrenocortical carcinoma; BLCA, Bladder Urothelial Carcinoma; BRCA, Breast invasive carcinoma; CESC, Cervical squamous cell carcinoma and endocervical adenocarcinoma; CHOL, Cholangiocarcinoma; COAD, Colon adenocarcinoma; DLBC, Lymphoid Neoplasm Diffuse Large B-cell Lymphoma; ESCA, Esophageal carcinoma; GBM, Glioblastoma multiforme; HNSC, Head and Neck squamous cell carcinoma; KICH, Kidney Chromophobe; KIRC, Kidney renal clear cell carcinoma; KIRP, Kidney renal papillary cell carcinoma; LAML, Acute Myeloid Leukemia; LGG, Brain Lower Grade Glioma; LIHC, Liver hepatocellular carcinoma; LUAD, Lung adenocarcinoma; LUSC, Lung squamous cell carcinoma; MESO, Malignant Mesothelioma; OV, Ovarian serous cystadenocarcinoma; PAAD, Pancreatic adenocarcinoma; PCPG, Pheochromocytoma and Paraganglioma; PRAD, Prostate adenocarcinoma; READ, Rectum adenocarcinoma; SARC, Sarcoma; SKCM, Skin Cutaneous Melanoma; STAD, Stomach adenocarcinoma; TGCT, Testicular Germ Cell Tumors; THCA, Thyroid carcinoma; THYM, Thymoma; UCEC, Uterine Corpus Endometrial Carcinoma; USC, Uterine Carcinosarcoma. UVM, Uveal Melanoma. **b** KLK8 expression in LUAD and LUSC based on data in GEPIA. Left, LUAD. Right, LUSC. Red, tumor sample (T). Black, normal sample (N). Asterisk, *p* value< 0.05; Centerline, median; box limits, upper and lower quartiles; points, outliers. **c-f** K-M curve showing the overall survival of LUSC patients stratified by KLK8 expression in the TCGA LUSC data and GEO LUC datasets. Red, KLK8 low expression group. Green, KLK8 high expression group. TCGA LUSC data (**c**). GSE 73403 (**d**). GSE157011 (**e**). GSE30219 (**f**)

Using cBioportal for Cancer Genomics (https://www.cbioportal.org/), the pan-cancer analysis demonstrated that KLK8 was significantly highly expressed in the tumor tissues compared with the normal samples in multiple cancer types, including COAD (Colon adenocarcinoma), ESCA (Esophageal carcinoma), LUAD (Lung adenocarcinoma), LUSC (Lung squamous cell carcinoma), READ (Rectum adenocarcinoma), STAD (Stomach adenocarcinoma), and THCA(Thyroid carcinoma) (Fig. 1a). Focusing on lung cancer, KLK8 expression was remarkably upregulated in LUSC using GEPIA (http://gepia.cancer-pku.cn/) (Fig. 1b). To further validate the prognostic capability of KLK8 in LUSC, we examined the survival of patients with different KLK8 expressions in four public LUSC datasets and found that patients with higher KLK8 expression were with significantly shorter overall survival (TCGA LUSC cohort: HR = 1.02, 95% CI, 1.01–1.04, *p* = 0.0032; GSE73403: HR = 3.17, 95% CI, 1.39–7.21, *p* = 0.004. GSE15011: HR = 1.40, 95% CI, 1.04–1.90, *p* = 0.026; GSE30219: HR = 1.98, 95% CI, 1.03–3.80, *p* = 0.037.) (Fig. 1c, d, e, f).

### KLK8 is an independent risk factor for LUSC

**Fig. 2:**
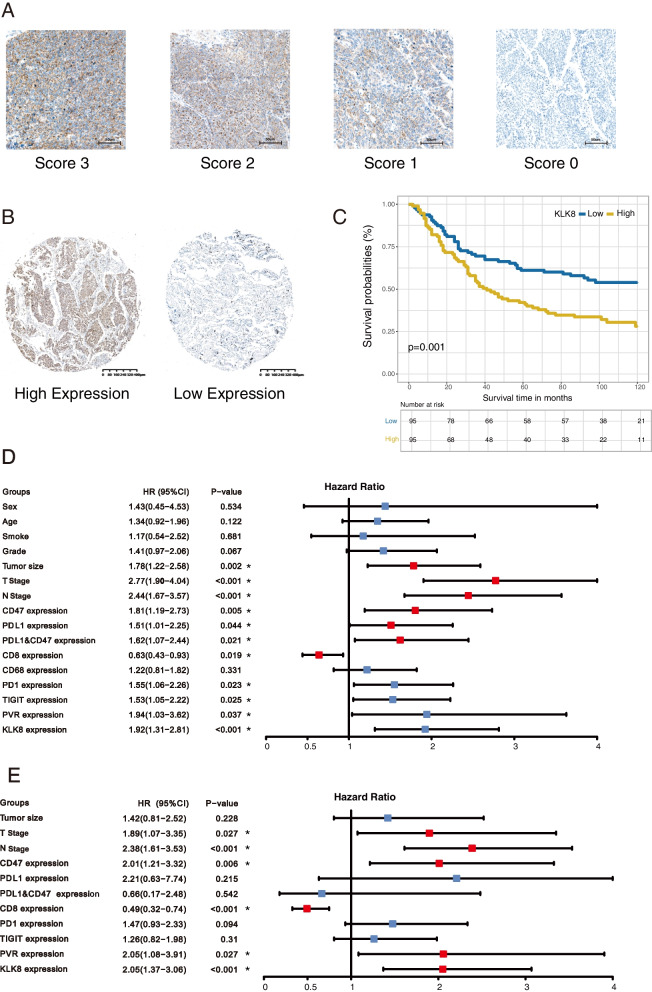
KLK8 expression in LUSC tumor microarray (TMA). **a** KLK8 staining intensity scoring standard. KLK8 expression was graded into 4 categories: 0, 1, 2, and 3 according to the staining intensity. The larger number represented higher KLK8 staining intensity (Magnification, 100X. Scale bar, 50 μm). The final score of samples is evaluated by multiplying the grade and corresponding area percentages. **b** Example of high and low KLK8 expression in TMA. Left, high expression sample. Right, low expression sample (Magnification, 20X. Scale bar, 400 μm). **c** K-M curve showing the overall survival in LUSC TMA stratified by KLK8 median expression. Blue, KLK8 low expression group. Yellow, KLK8 high expression group. **d** Univariate analysis forest plot of clinicopathological factors, KLK8 expression, and other immune markers’ expression in LUSC TMA. CoExp, PD-L1, and CD47 co-expression. CD8+ TILs, CD8+ tumor infiltration lymphocytes. CD68+ M, CD68+ infiltration macrophage infiltration level. The red square represents factors that were statistically significant for prognosis, while the blue square represents factors that were without statistical significance for prognosis. **e** Multivariate analysis forest plot of factors, which could predict the overall survival in the univariate analysis. CoExp, PD-L1, and CD47 co-expression. CD8 + TILs, CD8+ tumor infiltration lymphocytes. The red square represents factors that were statistically significant for prognosis, while the blue square represents factors that were without statistical significance for prognosis

To further validate the prognostic role of KLK8, we introduced a LUSC FFPE microarray (*n* = 190), of which the patients were retrospectively recruited in the Department of Thoracic Surgery, National cancer center (Beijing, China), into our study. The clinicopathological information of this microarray cohort was in Table 1. Based on the 8th AJCC (American Joint Committee on Cancer) Staging Manual [29], the number and percentage of each stage were as follows: 34 (17.89%) for stage I patients, 70 (36.84%) for stage II patients, and 86 (45.26%) for stage III patients at diagnosis. The median follow-up time was 66.5 months. At the last follow-up, 78 (41.05%) patients were alive or censored. We performed the KLK8 IHC on this microarray and quantified the results. The representative images of IHC score criteria and KLK8 expressions were displayed (Fig. 2a, b). The median IHC score of KLK8 is 1.235, and we classified the samples into high and low KLK8 expression subgroups based on the median score as the cut-off value. Then we conducted the survival analysis and found that high KLK8 expression was significantly associated with inferior prognosis (*p* = 0.001, Fig. 2c). Further, we performed univariate Cox regression analyses (Fig. 2d, Table 2) and multivariate Cox regression analyses (Fig. 2e, Table 3) for LUSC-associated potential factors and identified that high KLK8 expression was an independent risk factor for LUSC patients in both the two Cox regressions (Univariate, HR =1.92, 95% CI, 1.31–2.81, *p*-value< 0.001; Multivariate, HR =2.05, 95% CI, 1.37–3.06, *p* value< 0.001). The KLK8 score and clinical information of each sample in our cohort were recorded in Supplementary Data 2.

**Table 1:** Clinicopathological characteristics of SqCLC patients in tissue microarray

Characteristics	Groups	Number of patients	Percentage of patients(%)
**Gender**	Male	183	96.32
	Female	7	3.68
**Age**	≤ 60 years old	88	46.32
	> 60 years old	102	53.68
**Smoking**	Yes	176	92.63
	No	14	7.37
**T stage**	T1	25	13.16
	T2	99	52.11
	T3	45	23.68
	T4	21	11.05
**N stage**	N0	64	33.68
	N1	67	35.26
	N2	59	31.05
**TNM stage**	I	34	17.89
	II	70	36.84
	III	86	45.26

**Table 2:** Univariate Cox regression of prognostic factors in SqCLC

	OS	
	HR (95%CI)	***p***-value
**Sex**		
Female	1.000	
Male	1.43 (0.45–4.53)	0.534
**Age**		
≤60	1.000	
> 60	1.34 (0.92–1.96)	0.122
**Smoking**		
No	1.000	
Yes	1.17 (0.54–2.52)	0.681
**Grade**		
Low	1.000	
Mid-high	1.41 (0.97–2.06)	0.067
**Tumor size**		
≤5	1.000	
> 5	1.78 (1.22–2.58)	0.002
**T stage**		
T1 + T2	1.000	
T3 + T4	2.77 (1.9–4.04)	< 0.001
**N Stage**		
N0 + N1	1.000	
N2	2.44 (1.67–3.57)	< 0.001
**CD47 expression**		
Low	1.000	
High	1.81 (1.19–2.73)	0.005
**PDL1 expression**		
Low	1.000	
High	1.51 (1.01–2.25)	0.044
**PDL1&CD47 expression**		
Low	1.000	
High	1.62 (1.07–2.44)	0.021
**CD8 expression**		
Low	1.000	
High	0.63 (0.43–0.93)	0.019
**CD68 expression**		
Low	1.000	
High	1.22 (0.81–1.82)	0.331
**PD1 expression**		
Low	1.000	
High	1.55 (1.06–2.26)	0.023
**TIGIT expression**		
Low	1.000	
High	1.53 (1.05–2.22)	0.025
**PVR expression**		
Low	1.000	
High	1.94 (1.03–3.62)	0.037
**KLK8 expression**		
Low	1.000	
High	1.92 (1.31–2.81)	< 0.001

All *p* values were two sides and less than 0.05 were considered significant. *SqCLC* Squamous cell lung cancer, *OS* overall survival, *HR* hazard ratio, *CI* confidence interval. Low and high expression was classified as the median except for CD47 (1% as cut-off value)

**Table 3:** Multivariate Cox regression of prognostic factors in SqCLC

	OS	
	HR (95%CI)	***p***-value
**Tumor size**		
≤5	1.000	
> 5	1.42 (0.81–2.52)	0.228
**T**		
T1 + T2	1.000	
T3 + T4	1.89 (1.07–3.35)	0.027
**N**		
N0 + N1	1.000	
N2	2.38 (1.61–3.53)	< 0.001
**CD47**		
Low	1.000	
High	2.01 (1.21–3.32)	0.006
**PDL1**		
Low	1.000	
High	2.21 (0.63–7.74)	0.215
**PDL1&CD47 expression**		
Low	1.000	
High	0.66 (0.17–2.48)	0.542
**CD8 expression**		
Low	1.000	
High	0.49 (0.32–0.74)	< 0.001
**PD1 expression**		
Low	1.000	
High	1.47 (0.93–2.33)	0.094
**TIGIT expression**		
Low	1.000	
High	1.26 (0.82–1.98)	0.31
**PVR expression**		
Low	1.000	
High	2.05 (1.08–3.91)	0.027
**KLK8 expression**		
Low	1.000	
High	2.05 (1.37–3.06)	< 0.001

All *p* values were two sides and less than 0.05 were considered significant. *SqCLC* Squamous cell lung cancer, *OS* overall survival, *HR* hazard ratio, *CI* confidence interval. Low and high expression was classified as the median except for CD47 (1% as cut-off value)

### Prognostic model underpinned by KLK8 in LUSC patients

**Fig. 3:**
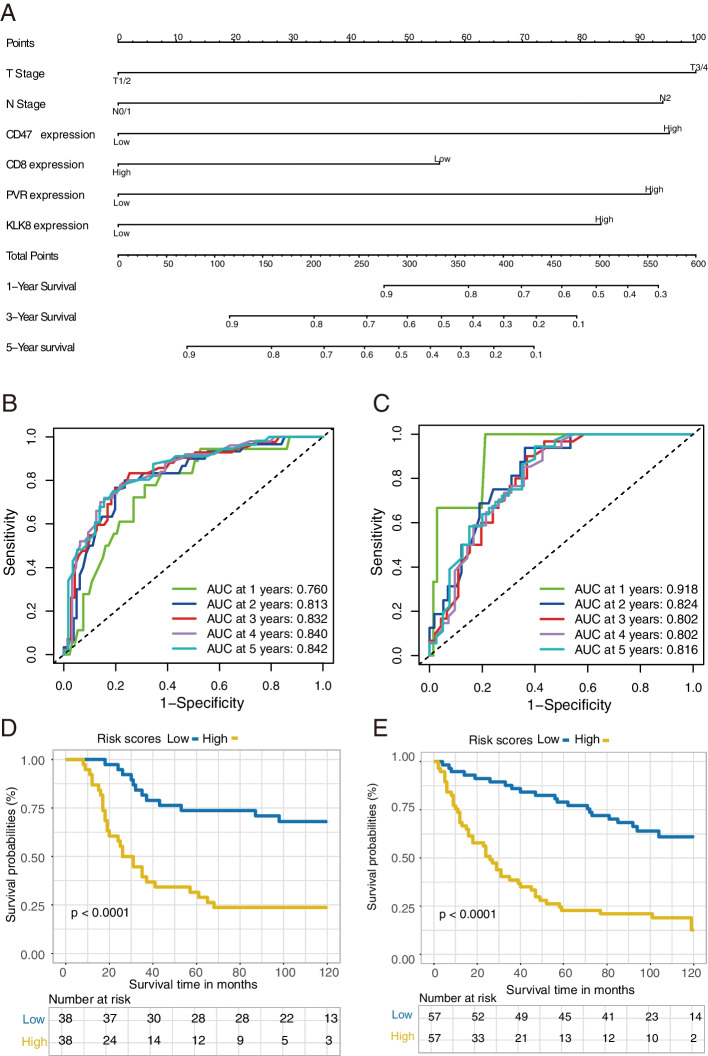
KLK8 expression-based prognosis prediction model of LUSC. **a** Prognostic nomogram based on the clinicopathological factors, KLK8 expression, and other immune markers predicting the 1-year, 2-year, and 3-year overall survival rate in our LUSC cohort. T, T stage. N, N stage. CoExp, PD-L1, and CD47 co-expression. CD8TILs, CD8+ tumor infiltration lymphocytes. CD68 Macrophage, CD68+ infiltration macrophage. **b-c** Receiver Operating Characteristic (ROC) curves of the prognostic nomogram in the training set (**b**) and the testing set (**c**). **d-e** K-M curves showing the overall survival in LUSC TMA stratified by the prognostic nomogram in the training set (**d**) and the testing set (**e**). Blue, KLK8 low expression group. Yellow, KLK8 high expression group

To further explore the survival-predicting role of KLK8 in LUSC, we established a prognostic model using the multivariate Cox regression model based on the LUSC cohort from our center (*n* = 190). All clinicopathological factors and immune marker expression status were included. The nomogram evaluating the 1-year, 3-year, and 5-year survival data of each patient was displayed (Fig. 3a) and the regression formula was “1.0677*T + 0.9634*N + 0.6733*CD47-CD8*0.3057 + PVR*0.8723 + KLK8*0.7273”. Total points over 440 could predict a survival rate of < 70% in 1-year survival, a survival rate of < 20% in 2-year survival, and a survival rate of < 10% in 3-year survival. To examine the sensitivity and specificity of this model, the LUSC cohort (n = 190) patients were randomly divided into the training set and the testing set. We found that the risk score calculated by the model emerged as a significant predictor for patients’ survival with high sensitivity and specificity (Fig. 3b, c). Survival analysis validated the robust prognostic ability of the predictor model in both patient sets (Fig. 3d, e).

### Exploration of KLK8-related function through pathway enrichment

**Fig. 4:**
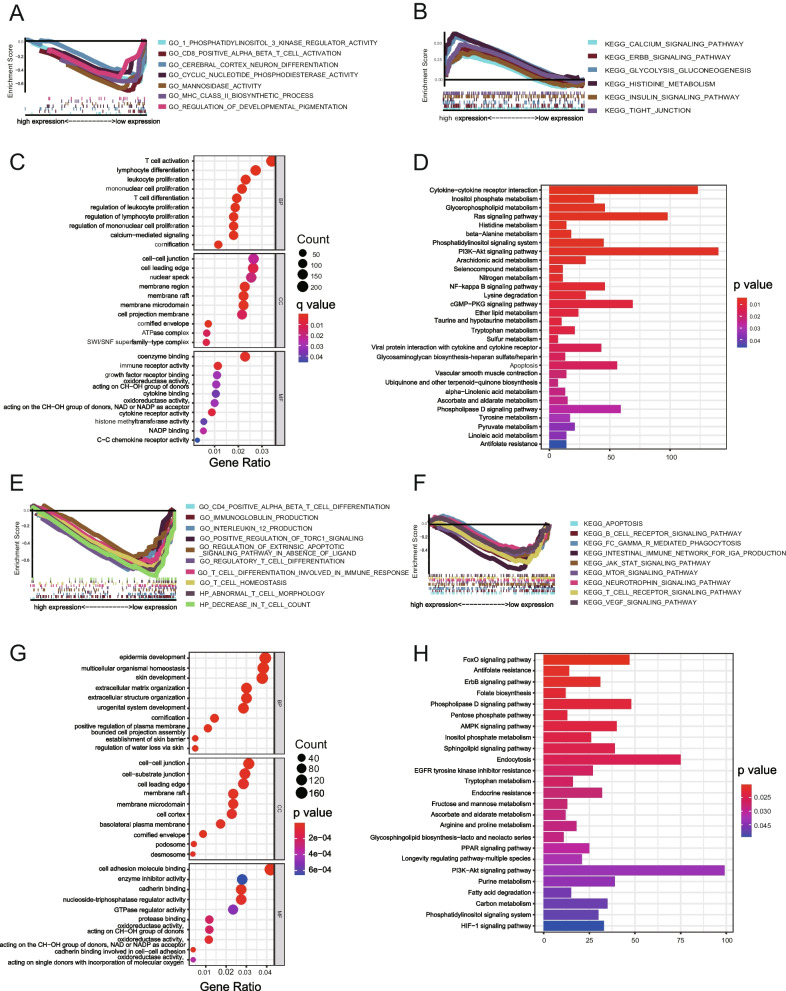
Pathway enrichment of patients with different KLK8 expression levels in LUSC public data. **a-b** Representative pathways highly enriched in KLK8 low expression patients (**a**) and KLK8 high expression patients (**b**) based on the GEO LUSC datasets (GSE73403, GSE15011, GSE3021). **c** Representative GO terms and pathways enriched in DEGs between high and low KLK8 expression groups based on the GEO LUSC datasets (GSE73403, GSE15011, GSE3021). **d** Representative KEGG [25–27] terms and pathways enriched in DEGs between high and low KLK8 expression groups based on the GEO LUSC datasets (GSE73403, GSE15011, GSE3021). **e-f** Representative pathways highly enriched in KLK8 low expression patients (**a**) and KLK8 high expression patients (**b**) based on the TCGA LUSC data. **g** Representative GO terms and pathways enriched in DEGs between high and low KLK8 expression groups based on the TCGA LUSC data. **h** Representative KEGG [25–27] terms and pathways enriched in DEGs between high and low KLK8 expression groups based on the TCGA LUSC data

Currently, the underlying mechanisms by which KLK8 promotes LUSC progression remain unsettled. To address this issue, we performed GO/KEGG pathway enrichment based on the DEGs derived from LUSC patients with different KLK8 expressions in the GEO LUSC datasets (GSE73403, GSE15011, GSE30219) (Fig. 4a, b, c, d) and TCGA LUSC dataset (Fig. 4e, f, g, h), respectively. Based on the GEO dataset, we found that the MHC Class II-related pathway and the CD8 T cell activation-related pathway were enriched in patients with low KLK8 expression and they were downregulated in patients with high KLK8 expression, indicating that low KLK8 expression might be associated with a more active immune microenvironment, which was favorable for tumor elimination. Moreover, the GO analyses showed that the T cell activation, lymphocyte differentiation, leukocyte proliferation, and cytokine-cytokine receptor interaction were mainly enriched in the biological processes (BP), suggesting the close relationships between KLK8 and cancer immunity. Likewise, based on the TCGA LUSC cohorts, we found that several immune-related pathways were enriched in patients with high KLK8 expression, such as the CD4 + T cell differentiation pathway, immunoglobulin production pathway, IL-12 production pathway, T cell homeostasis, and differentiation pathway, and T cell receptor signaling pathway. More evidence about the interactions between KLK8 expression and cancer immunity warrants further exploration.

### High KLK8 expression promotes suppressive TIME in LUSC

**Fig. 5:**
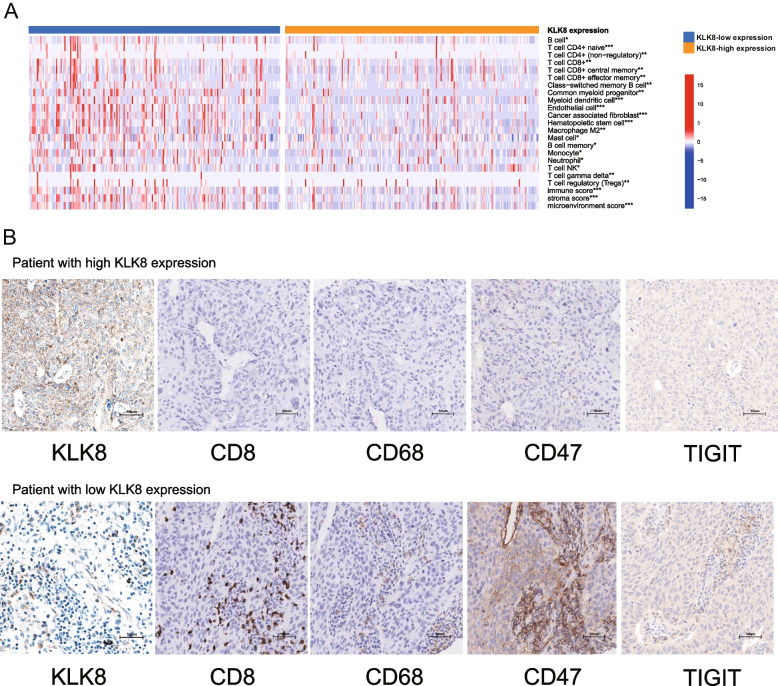
Tumor microenvironment (TME) features of patients with different KLK8 expressions. **a** The immune cell infiltration spectrum of patients with different KLK8 expressions. **b** Immunohistochemistry (IHC) results of immune markers in representative patients from the LUSC TMA. Upper, representative patient with high KLK8 expression. Lower, representative patient with low KLK8 expression

Considering that several immune-related pathways were enriched above, we then characterized the TIME status of patients with high/low KLK8 expressions. Using the TCGA LUSC cohort, we profiled the immune cell infiltration spectrum of patients with different KLK8 expressions (Fig. 5a). Most cell types showed significantly higher infiltration levels in KLK8 low expression group, including CD4 + T cells, CD8 + T cells, and dendritic cells. While some immunosuppressive cells with cancer-promoting nature, like regulatory T cells (Tregs), M2 macrophages, and cancer-associated fibroblasts, displayed more intense infiltration in the KLK8 low expression group. These results indicate that high KLK8 expression might be important in shaping the immune-suppressive TIME in LUSC, and this conclusion was supported by some patients from the LUSC cohort (*n* = 190). In Fig. 5b, we showed the IHC images of two representative patients with different KLK8 expressions, respectively. The patient with high KLK8 expression was with negative expression of immune markers, including CD8, CD68, CD47, and TIGIT. While the patient with low KLK8 expression was with abundant expression of these immune markers.

### Association between KLK8 and immune checkpoints and immune cells

**Fig. 6:**
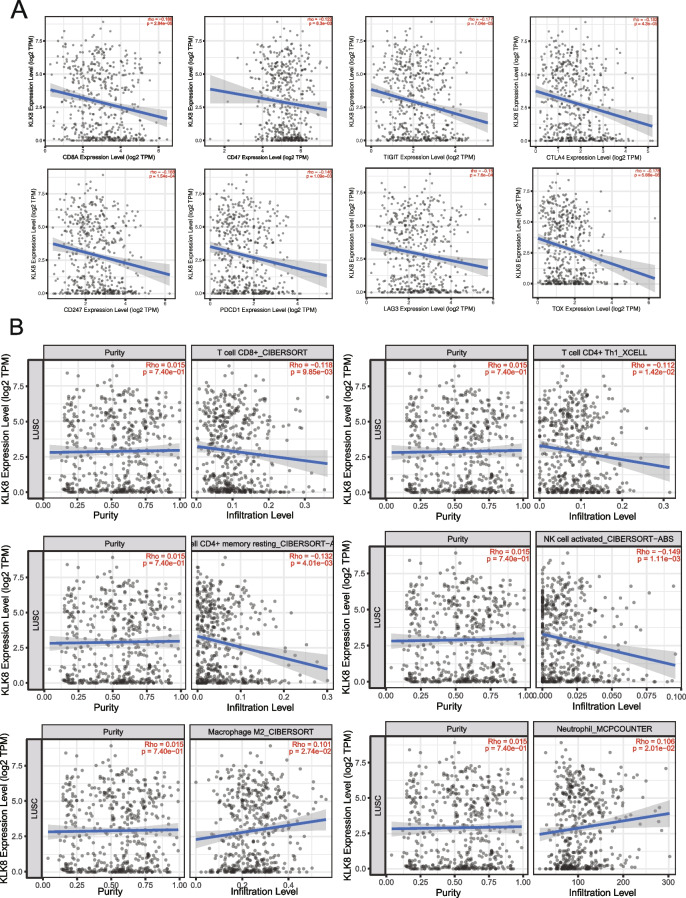
Associations between KLK8 expression and classical immune markers/canonical immune cells. **a** Correlations between KLK8 expression and CD8A, CD47, TIGIT, CTLA4, CD247 (PD-L1), PDCD1 (PD-1), LAG3, and TOX. **b** Correlations between KLK8 expression and CD8+ Tcells, CD4+ T cells, natural killer (NK) cells, macrophages, and neutrophils

To further explore the potentiality of KLK8 as a target in LUSC immunotherapy, we evaluated the associations between KLK8 and several immune checkpoints, immune markers, and immune cells in LUSC using TIMER (http://timer.cistrome.org). We observed that KLK8 was negatively correlated with CD247, PDCD1, CTLA4, CTLA4, LAG3, TIGTI, and TOX, and all these correlations were significant statistically (Fig. 6a). In addition, KLK8 expression is negatively correlated with cancer-killing effector cells, such as CD8 + T cells which repress cancer progression by the direct killing effects, and CD4+ Th1 cells which enhance the cytotoxic activity of CD8 + T cells. Meanwhile, immunosuppressive cells, such as the M2 macrophages and neutrophils were positively correlated with KLK8 expression (Fig. 6b). Collectively, these results suggested that KLK8 had the potency to be an immune target in LUSC, while the detailed mechanism needs further research.

## Discussion

In this study, we revealed that KLK8 was a novel prognostic indicator of LUSC in public databases and validated this prognosis capacity in the LUSC cohort (*n* = 190) from our center. In addition, we demonstrated the close associations between KLK8 and cancer immunity and showed the potentiality of KLK8 as a therapeutic target in LUSC.

KLK8 belongs to the serine protease kallikrein subfamily, capable of degrading a series of proteins, such as fibronectin, which is a ubiquitous extracellular matrix (ECM) glycoprotein [30]. Currently, most KLK members have been confirmed to be regulators and targets in cancer immunity, and they were mainly reported as cancer-promoting factors [10]. For instance, the prostate-specific antigen (kallikrein-3) and kallikrein 2 are representative biomarkers in prostate cancer [8], promoting prostate cancer progression through activating malignant cell proliferation invasion, and extracellular matrix (ECM) degradation [31]. However, the research about KLK8 was insufficient and contrary opinions exist on how KLK8 behaves in cancer. Previous studies have reported that KLK8 was a prognostic factor in various cancer types, including cervical cancer [13], lung cancer [22], and ovarian cancer [11]. Hua, Q et al. found that KLK8 promoted cancer cell proliferation and inhibited apoptosis by activating the PI3K-AKT-mTOR signaling pathway in pancreatic carcinoma [16]. In colorectal cancer, KLK8 facilitated the EMT processes which could be mitigated by protease-activated receptor antagonists, suggesting that the protease activity might be the mechanism by which KLK8 exerted its tumor-promoting functions [16]. KLK8 was also involved in the progression of melanoma [32], which further suggested the tumor-promoting role of KLK8. However, some studies reported that KLK8 was a favorable prognostic marker in cancer. In ovarian cancer, previous researchers reported that patients with human kallikrein 8-positive tumors had significantly longer survival and a lower risk of relapse/death [12, 33].

In NSCLC, the functions of KLK8 were not always consistent. The KLK8 transcript 1 and KLK8 transcript 2 were against tumor cell dissemination, while the KLK8 transcript 4 was on the opposite [9]. Collectively, the functions of KLK8 are context-dependent, especially in lung cancer. Little is known about how KLK8 behaves in LUSC before our study. Using public data and the FFPE microarray of our LUSC cohort, we confirmed the prognostic capacity of KLK8, setting the tone of KLK8 function in LUSC and paving the way for the subsequent KLK8 research.

In recent decades, checkpoint inhibitor-based immunotherapy has substantially improved survival to NSCLC [34]. Although several ICI-based clinical trials have been conducted in LUSC [7], the efficacy of immunotherapy in LUSC is not satisfying [35]. Therefore, exploring the novel immune target in LUSC is worthy of attention. Previous studies have reported that kallikrein-related proteins could participate in cancer immunity regulation. For instance, KLK3 could promote immunosuppression by upregulating TGF-β [8], and KLK5 can cleave chemo-attractant molecules, such as cathelicidin, to disturb immune defense [36]. KLK4 and KLK3 are also reported to trigger cytotoxic T-cell activation as an immunogenic molecule [37, 38]. However, little was known about the significance of KLK8 in cancer immunotherapy before our study. Compared to the patients with high KLK8 expression, we found that several immune-related signaling pathways were enriched in the patients with low KLK8 expression, and they have a more active immune cell infiltration status, suggesting the potentiality of KLK8 as a novel therapeutic target in LUSC.

Regarding the remarkable difference in the intratumoral infiltration of diverse immune cells between high and low KLK8 expression subgroups, we speculate that the immunosuppressive effects of KLK8 may derive from the regulatory impact on chemotactic protein production, remodeling, and degradation. The reshaping of ECM is implicated in regulating immune cell accessibility [39]. In melanoma, KLK8, TIGIT, and TRIM63 were identified as the representative three-gene classifier for melanoma molecular subtypes to predict patients’ survival risk. Melanoma patients with low KLK8 and high TIGIT were classified as the “Immune” subtype, a molecular subtype with favorable survival [40]. Our study also identified the negative correlation between KLK8 and TIGIT expression. TIGIT is a novel immunotherapy target [41], which has been identified as an immunosuppressive molecule through repressing effector anti-tumor immune cells in the cancer-immune microenvironment [42].

Limitations existed. Our study identified a KLK8-based single-gene model predicting patients’ survival. Single-gene models are inferior in stability and accuracy compared to the pathway-associated signature. A large sample size is demanded to validate the efficacy of the single-gene model. However, the predictive capacity of our model is still significant, and the single-gene model has also been reported by previous study [43]. Compared to other prognostic models using single or a few individual genes [44–46], KLK8 was more significantly associated with LUSC patients’ survival, which was validated in our in-house cohort and several public LUSC datasets. In addition, our multivariate regression formula gave a more exact estimation of patients’ survival based on clinical stages and marker expressions simply measured via IHC.

In summary, we reported the predictive ability and the extensive anti-tumor immunomodulatory capacity of KLK8 in LUSC, suggesting the potentiality of KLK8 as a novel immunoregulatory target in LUSC. Over the last two decades, many researchers have contributed to exploring KLK inhibitors [47]. LeBeau et al. reported compounds that decreased the KLK3 level in prostate cancer cell lines and in human prostate cancer xenograft [48]. The approval of Gleevec, which was the first kinase inhibitor, introduced the era of using genomic data for targeted therapies in oncological treatment. However, only a few kinomes have been effectively “drugged” so far [49]. KLK8, a member of the Kallikrein series, is a promising candidate for target.

Considering our findings, we are looking forward to the introduction of KLK8 inhibitors and the combinational usage of KLK8 inhibitors and ICIs in LUSC.

### Supplementary Information

**Additional file 1:** **Supplementary Data 1.** Immune infiltration within the tumor microenvironment for low-KLK8 and high-KLK8 patient groups.

**Additional file 2:** **Supplementary Data 2.** The clinicopathological data of Tissue Microarrays.

## Data Availability

The original contributions presented in the study are included in the article/Supplementary Material. Further inquiries can be directed to the corresponding authors.
